# Chest CT in COVID-19 pneumonia: a correlation of lung abnormalities with duration and severity of symptoms

**DOI:** 10.1186/s43055-020-00359-z

**Published:** 2020-12-01

**Authors:** Mohammed Mahmoud Dawoud, Tamer Mahmoud Dawoud, Noha Yousef Amin Ali, Hanan Ahmad Nagy

**Affiliations:** 1 Radiodiagnosis & Medical Imagining, Faculty of Medicine, Tanta University, El-geish Street, Tanta, Gharbya Governorate Egypt; 2 Radiodiagnosis & Medical Imagining, Faculty of Medicine, Tanta University, El-geish street, Tanta, Gharbya Governorate Egypt; 3 Clinical pathology, Faculty of Medicine, Tanta University, El-geish street, Tanta, Gharbya Governorate Egypt; 4 Radiodiagnosis and Medical Imaging, Faculty of Medicine, Tanta University, El-geish Street, Tanta, Gharbia governorate Egypt

**Keywords:** Coronavirus, COVID-19 pneumonia, Computed tomography, Duration and severity, Ground-glass opacity, PCR

## Abstract

**Background:**

After the spread of COVID-19 pneumonia, chest CT examination was used as a substantial non-invasive complement to RT-PCR for diagnosing and as a rapid screening tool when RT-PCR results are unavailable. Our study aimed at the analysis of the lung abnormalities detected by chest CT in COVID-19 pneumonia according to the severity and duration of symptoms.

**Results:**

In the early phase (*n* = 60), 32 patients had negative CT findings and 28 patients had positive findings with a mean total lung severity score of 2.13. In the intermediate phase (*n* = 116), 4 patients had negative CT findings and 112 patients had positive findings with a mean total lung severity score of 16.08. In the late phase (*n* = 36), all patients had positive findings with a mean total lung severity score of 17.83. CT lung abnormalities were progressed on follow-up CT studies. We found a high total lung severity score in many patients with mild symptoms with a mean of 14.77 and a low total lung severity score in many patients with moderate to severe symptoms with a mean of 9.14.

**Conclusion:**

Chest CT should be used as a routine examination for diagnosing COVID-19 pneumonia and follow-up of disease advance. The progression of lung abnormalities was related to the duration more than the severity of symptoms.

## Background

In December 2019, a novel coronavirus disease 2019 (COVID-19) was identified in Wuhan, Hubei Province, China. On January 30, 2020, the COVID-19 outbreak had been pronounced by the World Health Organization (WHO) as a Public Health Emergency of International Concern, and furthermore, a pandemic on March 11, 2020. From April 8, 2020, this epidemic had spread out to more than 199 countries affecting more than one million persons worldwide with 81,478 reported deaths [1–3]. By June 30, 2020, Egypt recorded 65188 coronavirus cases since the epidemic began, according to the World Health Organization (WHO) [4]. In addition, Egypt reported 2708 coronavirus deaths. However, these numbers are possibly disregarded as not all patients are tested, particularly asymptomatic patients, or those with mild symptoms and no associated comorbidities [5].

Coronavirus 2019 (COVID-19) is the seventh known coronavirus that achieves human-to-human transmission despite its zoonotic origin causing serious coronavirus disease (COVID-19), mainly pneumonia [6–8]. Similar pulmonary syndromes have been caused by other strains of the coronavirus family as the severe acute respiratory syndrome (SARS), with no human infection reported since 2003, and the Middle East respiratory syndrome (MERS), with small outbreaks, continue to be reported [8–10].

As regards the current epidemiological surveys, the incubation period of this disease is 1–14 days, mostly 3–7 days. Fever, dry cough, and fatigue are the most common clinical symptoms at presentation in addition to other symptoms such as nasal obstruction, runny nose, sore throat, dyspnea, myalgia, and diarrhea. About 20% of patients have a serious illness that may rapidly progress to ARDS with a 3% mortality rate [11–13].

Confirming COVID-19 infection is based on microbiological tests such as real-time polymerase chain reaction (RT-PCR) which is routinely used to detect causative viruses of respiratory secretions obtained by bronchoalveolar lavage, endotracheal aspirate, nasopharyngeal swab, or oropharyngeal swab [14, 15].

However, the RT-PCR test has limitations; improper sampling site of the swab test and scanty viral material in the specimen or procedural error can cause false-negative results. So, the RT-PCR test for COVID-19 has high specificity but low sensitivity as reported (59–71%). Also, the RT-PCR process is time-consuming and might not be available in an emergency circumference and their results are not immediately available. These limitations may delay medical isolation and the transmission of infection [16, 17].

To compensate for those shortcomings, CT examination, with its high resolution, can be used as an important non-invasive complement to RT-PCR for diagnosing COVID-19 pneumonia in the current outbreak. CT equipment is widespread and the CT scan process is simple and quick, it can be used as a screening tool for suspected patients in the severe epidemic center with the unavailability of RT-PCR results. It is believed that in emergencies, patients with positive CT findings suggesting COVID-19 should be first isolated and then subjected to RT-PCR testing to confirm the diagnosis [16, 17].

Also, according to the recent reports, lung CT imaging may manifest abnormalities earlier than RT-PCR; when RT-PCR is negative especially the initial one in suspected patients, atypical suggestive CT abnormalities could be found. So, CT is more sensitive than RT-PCR and can decrease the chance of false-negative results in the RT-PCR assay [18, 19].

Different studies described various CT chest manifestations of COVID-19 pneumonia; it was found that the main CT feature of COVID-19 pneumonia is ground-glass opacities (GGO), typically with bilateral involvement affecting multiple lobes, particularly the lower lobes and a peripheral and sub-pleural distribution. This may be associated with areas of focal consolidation or thickened intralobular reticulations giving a crazy-paving appearance. Also, signs of resolving pneumonia could be seen in patients several days after disease onset as a reverse halo sign; areas of ground-glass surrounded by peripheral consolidation. Pleural effusion, cavitation, pulmonary nodules, pneumothorax, and lymphadenopathy are rare chest findings in case of COVID-19 pneumonia [20–22].

The aim of this study is to correlate lung abnormalities detected by thin-section chest CT in COVID-19 pneumonia with duration and severity of symptoms.

## Methods

### Study population

In this prospective study, non-contrast thin-section chest CT examinations were performed for 247 symptomatic patients suspected to have COVID-19 pneumonia throughout a period extending from May 17, 2020, to June 14, 2020.

Approval of the Research Ethics Committee (REC) and informed consent were obtained from all participants in this study after an explanation of the benefits and risks of the procedure. Privacy and confidentiality of all patients’ data were guaranteed. All data provisions were monitored and used for scientific purposes only.

The included criteria were symptomatic patients suspected to have COVID-19 pneumonia; contact with known positive COVID-19 patients, or presence of positive laboratory indicators (decreased lymphocytes, elevated CRP, elevated ESR), or with confirmed diagnosis by real-time RT-PCR. No sex or age predilection.

Exclusion criteria were patients who did not know the date of onset of symptoms and critical cases that were admitted to the intensive care unit for mechanical ventilation.

**Fig. 1:**
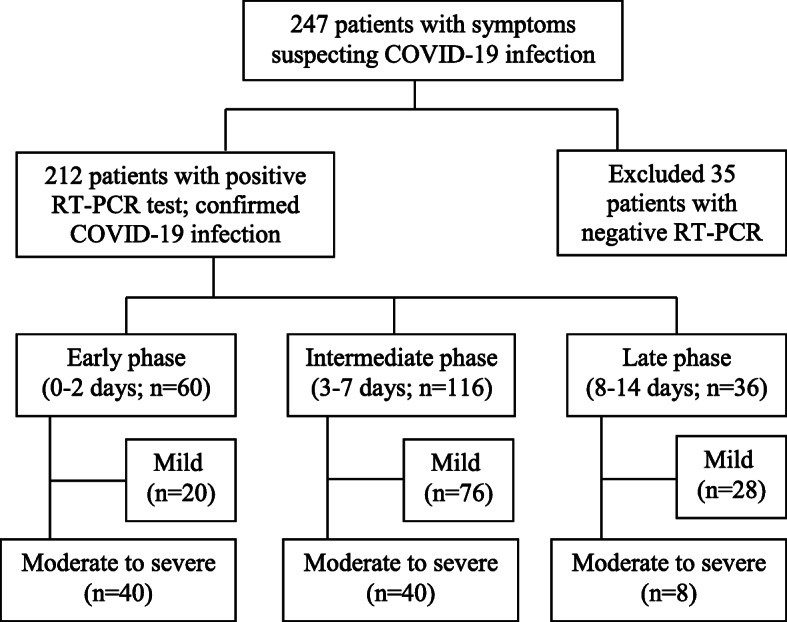
Flowchart of patient selection

After accepting RT-PCR as a standard reference for confirming COVID-19 infection, 35 patients with negative CT and RT-PCR (2 swabs, 5 days in-between) were excluded from the study and the 212 patients with confirmed COVID-19 infection by RT-PCR were included in the study (Fig. 1).

All the included participants were subjected to the following:

### Data collection

- Full medical history, including name, age, sex, history of contact to known positive COVID-19 patients, and present history of current symptoms: onset, course, and duration.
- Available recent laboratory investigation as complete blood count (CBC), C-reactive protein (CRP), erythrocyte sedimentation rate (ESR), lactate dehydrogenase (LDH), and D-dimer.
- Among the examined 247 symptomatic patients, 153 patients had available RT-PCR test; 119 of them were positive and 34 were negative. The patients with unavailable RT-PCR (94 patients) did it, and the patients with negative initial one repeated the test (5 days in-between) for confirmation. 35 symptomatic subjects had negative RT-PCR tests and 212 patients had positive RT-PCR tests.

The patients with positive RT-PCR were categorized:

- According to the duration of the symptoms (time between the onset of symptoms and initial CT examination) into 3 groups: early phase (0–2 days), intermediate phase (3–7 days), and late phase (8–14 days).
- According to the severity of clinical presentation into 2 groups: mild group (with mild symptoms of respiratory infection as fever and cough) and moderate to severe group (with dyspnea; respiratory rate ≧ 30 times per minute or oxygen saturation ≦ 93% at rest) depending on the clinical stages of COVID-19 issued by China and WHO interim guidance [23, 24].

### Computed tomography (CT) examinations

CT was performed using Toshiba Aquilion 16.

### CT protocol

- CT scans were performed at the end-inspiration level with patients in supine position and arms raised with 1.25-mm-section thickness for reconstruction, 1.25-mm gap, tube voltage 120 kV with automatic tube current modulation, DFOV 40.0 × 45.0 cm.

### Image analysis

- All the CT images of all patients were analyzed by two radiologists with 14 and 10 years of chest CT experience independently, blinded to the clinical data, and laboratory indicators, in a standard clinical Picture Archiving and Diagnostic System workstation, and final decisions reached by consensus are reported.
- Each CT scan was evaluated for the following criteria: the presence of chest abnormalities as GGO, focal consolidation, crazy paving, reversed halo, linear consolidation, pleural effusion, and others, and its distribution (peripheral, central, diffuse), laterality of involvement, number of affected lobes, and degree of involvement in each lobe.
- According to Bernheim et al. [25], the total lung severity score was calculated for each patient according to the degree of involvement in each of the five lung lobes by summing the five lobe score (ranging from 0 to 20). No lobe involvement (0%) corresponded to a lobe score of 0, minimal involvement (1–25%) to a lobe score of 1, mild (26–50%) to a lobe score of 2, moderate (51–75%) to a lobe score of 3, and severe (76–100%) to a lobe score of 4. Then, the range of severity score, mean, and standard deviation was calculated for the patients of each subgroup.

### Statistical analysis

- The collected data were coded, processed, and analyzed using the SPSS (Statistical Package for Social Sciences) version 22 for Windows® (IBM SPSS Inc, Chicago, IL, USA).
- Data were tested for the normal distribution using the Shapiro-Wilk test. Qualitative data were represented as frequencies and relative percentages. Quantitative data were expressed as mean ± SD (standard deviation) and range.
- Comparisons among the groups were performed using one-way ANOVA and independent sample *t* test.
- Probability (*P* value): *P* value ˂ 0.05 was considered significant. *P* value ˃ 0.05 was considered insignificant.
- Using a positive RT-PCR as the standard reference, the sensitivity, specificity, and negative predictive value were calculated.

## Results

Two hundred twelve patients with confirmed COVID-19 infection by any positive RT-PCR test were enrolled in this study; positive initial PCR tests were detected in 183 patients (86.3%).

Among the studied patients, 128 were male (60%) and 84 were female (40%). Their ages ranged from 6 to 75 years with a mean of 40.06 and SD ± 17.08. The majority of the patients (100 patients representing 47%) were in the age group from 20 to 40. Demographic data of the studied 212 patients is illustrated in Table 1.

**Table 1:** Demographic data of the studied 212 patients

Age in years	Sex		
	Male 128 (60%)	Female 84 (40%)	Total 212
6 to ≤ 20	11 (5.2%)	4 (1.9%)	15 (7.1%)
20 to ≤ 40	52 (24.5%)	48 (22.6%)	100 (47.1%)
40 to ≤ 60	29 (13.7%)	24 (11.3%)	53 (25%)
More than 60 years	35 (16.6%)	9 (4.2%)	44 (20.8%)

Data are numbers of patients (percentages of the total number, 212)

The most prevalent symptom was fever in 143 patients (67.5%), 140 patients (66%) presented with cough, 88 patients (41.5%) with dyspnea, 80 patients (37.7%) with sore throat, 72 (34%) patients with fatigue, 36 patients (17%) with myalgia, 12 patients (5.7%) with runny nose, and 9 patients (4.2%) with diarrhea.

Most of the patients had decreased lymphocyte count (1.13 ± 0.58 × 10^9^/L), mildly elevated C-reactive protein (CRP, 36.6 ± 22.8 mg/L), and erythrocyte sedimentation rate (ESR, 27.3 ± 23.7 mm/h). Some patients had elevated alanine aminotransferase (ALT, 35.1 ± 32.3 U/L), aspartate aminotransferase (AST, 34.8 ± 19.1 U/L), total bilirubin (TBIL 8.3 ± 4.1 mg/dL), urea (4.0 ± 1.8 mmol/L), creatinine (62.4 ± 18.4 μmol/L), lactate dehydrogenase (LDH, 307.5 ± 185.9 U/L), and D-dimer (0.6 ± 0.38 mg/L).

Included patients were divided depending on the severity of symptoms into two groups: mild (124 patients) and moderate to severe (88 patients). According to the duration from onset of symptoms, included patients were divided into early phase 0–2 days (60 patients), intermediate phase 3–7 days (116 patients), and late phase 8–14 days (36 patients).

Among 60 patients presented during the early phase, 20 patients of them had mild symptoms and 40 patients had moderate to severe symptoms. Among 116 patients presented during the intermediate phase, 76 patients of them had mild symptoms and 40 patients had moderate to severe symptoms. Among 36 patients presented during the late phase, 28 patients of them had mild symptoms and 8 patients had moderate to severe symptoms (Fig. 1).

**Fig. 2:**
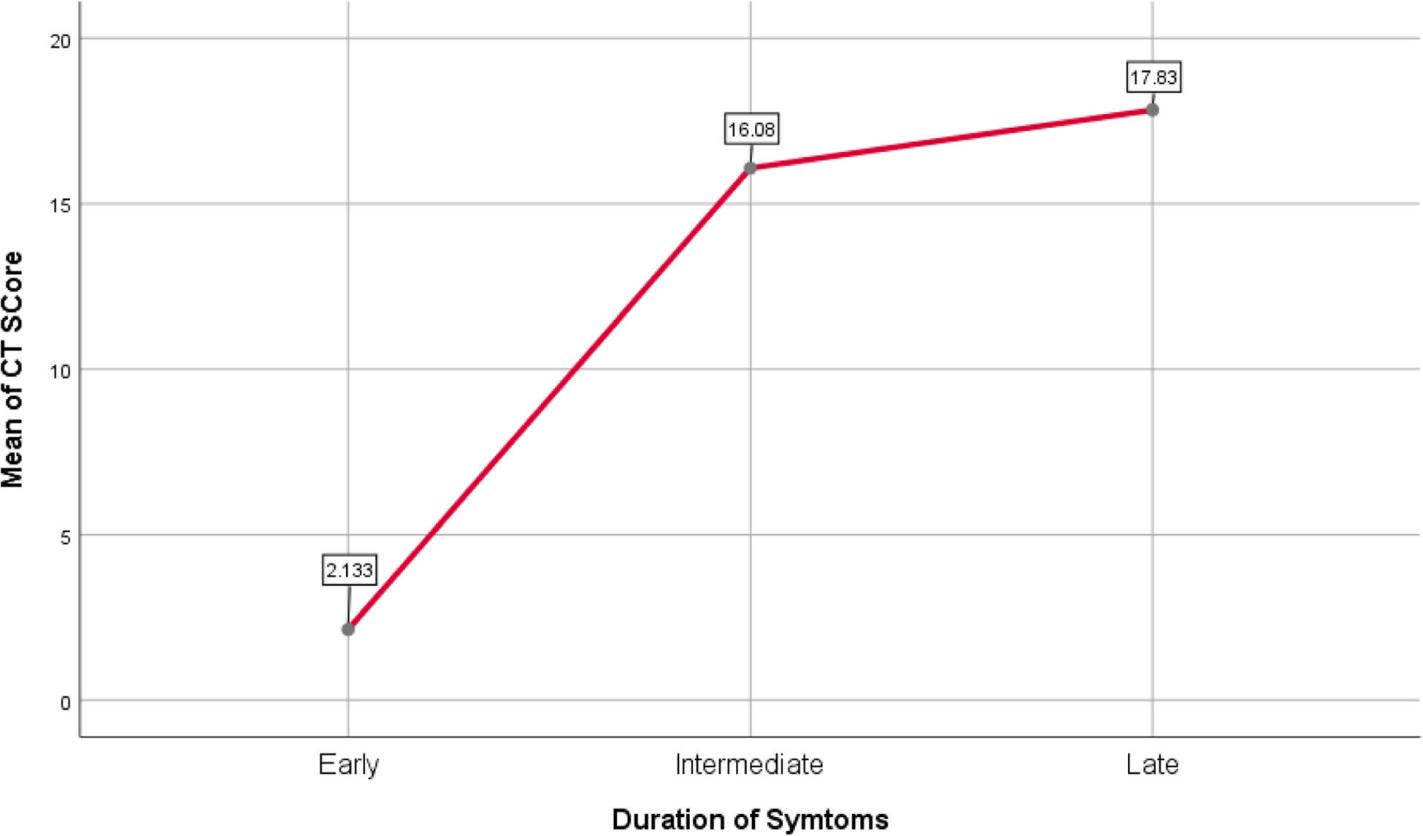
Relation between CT severity score and duration of symptoms

Non-contrast thin section chest CT was done for all patients. Initial CT revealed lung abnormalities in 176 patients with a mean of total lung severity score 12.43 ± 7.170, sensitivity 83%, and specificity 100%. The CT findings were analyzed in all patients and compared according to duration (*P* < 0.001) (Table 2, Fig. 2) and according to the severity of symptoms (*P* < 0.001) (Table 3).

**Table 2:** Distribution and frequency of the CT pulmonary abnormalities at different phases

	Early phase (0–2 days; *n* = 60)	Intermediate phase (3–7 days; *n* = 116)	Late phase (8–14 days; *n* = 36)
No CT findings	32 (15.1%)	4 (1.9%)	–
Positive CT findings	28 (13.2%)	112 (52.8%)	36 (17%)
GGO only	24 (11.3%)	9 (4.2%)	–
Consolidation only	–	12 (5.7%)	–
Both	4 (1.9%)	57 (26.9%)	36 (17%)
Crazy paving	–	32 (15.1%)	24 (11.3%)
Reversed halo	–	16 (%)	20 (9.4%)
Linear consolidation	–	–	28 (13.2%)
Pleural effusion	–	4 (1.9%)	–
Peripheral distribution	23 (10.8%)	24 (11.3%)	4 (1.9%)
Central distribution	–	–	–
Diffuse distribution	5 (2.3%)	88 (41.5%)	32 (15.1%)
Unilateral lung affection	4 (1.9%)	–	–
bilateral lung affection	24 (11.3%)	112 (52.8%)	36 (17%)
One lobe of affection	4 (1.9%)	–	–
Two lobes of affection	7 (3.3%)	–	–
Three lobes of affection	13 (6.1%)	28 (13.2%)	–
Four lobes of affection	4 (1.9%)	48 (22.6%)	13 (6.1%)
Five lobes of affection	–	35 (16.5%)	24 (11.3%)
Total lung severity score mean ± SD	2.13 ± 2.574	16.08 ± 3.141	17.83 ± 3.229

Data are numbers of patients (percentages of the total number, 212). *GGO* ground-glass opacity

**Table 3:** Distribution and frequency of the CT pulmonary abnormalities according to the severity of symptoms

	Mild symptoms (*n* = 124)	Moderate to the severe symptom (*n* = 88)
No CT findings	12 (5.7%)	24 (11.3%)
Positive CT findings	112 (52.8%)	64 (30.2%)
GGO only	12 (5.7%)	21 (9.9%)
Consolidation only	7 (3.3%)	5 (2.3%)
Both	56 (26.4%)	41 (19.3%)
Crazy paving	52 (24.5%)	4 (1.9%)
Reversed halo	32 (15.1%)	4 (1.9%)
Linear consolidation	22 (10.4%)	6 (2.8%)
Pleural effusion	4 (1.9%)	–
Peripheral distribution	33 (15.6%)	18 (8.5%)
Central distribution	–	–
Diffuse distribution	79 (37.3%)	46 (21.7%)
Unilateral lung affection	–	4 (1.9%)
bilateral lung affection	112 (52.8%)	60 (28.3%)
One lobe affection	–	4 (1.9%)
Two lobes affection	4 (1.9%)	3 (1.4%)
Three lobes affection	9 (4.2%)	32 (15.1%)
Four lobes affection	52 (24.5%)	13 (6.1%)
Five lobes affection	47 (22.2%)	12 (5.6%)
Total lung severity score mean ± SD	14.77 ± 5.93	9.14 ± 7.5

Data are numbers of patients (percentages of the total number, 212). *GGO* ground-glass opacity

In the early phase group (0–2 days, *n* = 60), 32 patients had negative CT findings and 28 patients had positive CT findings. CT abnormalities were GGO only in 24 patients, combined GGO and consolidation in 4 patients. Peripheral distribution of the pulmonary abnormalities was noted in 23 patients, and diffuse distribution was noted in 5 patients. The unilateral affection was detected in 24 patients with bilateral affection in 4 patients; one lobe affection in 4 patients, two lobes of affection in 7 patients, three lobes of affection in 13 patients, and 4 lobes of affection in 4 patients. According to the total lung severity score, 4 patients had a score of 2, 4 patients had a score of 3, 8 patients had a score of 4, 8 patients had a score of 6, and 4 patients had a score of 7. No crazy-paving, reversed halo signs, linear consolidation, or pleural effusion were detected during the early phase.

In the intermediate phase group (3–7 days, *n* = 116), 4 patients had negative CT findings and 112 patients had positive CT findings. CT abnormalities were GGO only in 9 patients, consolidation only in 12 patients, combined GGO and consolidation in 57 patients, crazy paving in 32 patients, reversed halo in 16 patients, and pleural effusion in 4 patients. Peripheral distribution of the pulmonary abnormalities was noted in 24 patients, and diffuse distribution was noted in 88 patients. Unilateral affection was detected in 4 patients with bilateral affection in 112 patients; 3 lobes of affection in 28 patients, 4 lobes of affection in 48 patients, and 5 lobes of affection in 35 patients. The total lung severity scores are as follows: score 13 in 4 patients, score 14 in 12 patients, score 15 in 17 patients, score 16 in 23 patients, score 17 in 32 patients, score 18 in 8 patients, and score 19 in 16 patients. No crazy-paving, reversed halo signs, linear consolidation, or pleural effusion were detected during the early phase.

**Fig. 3:**
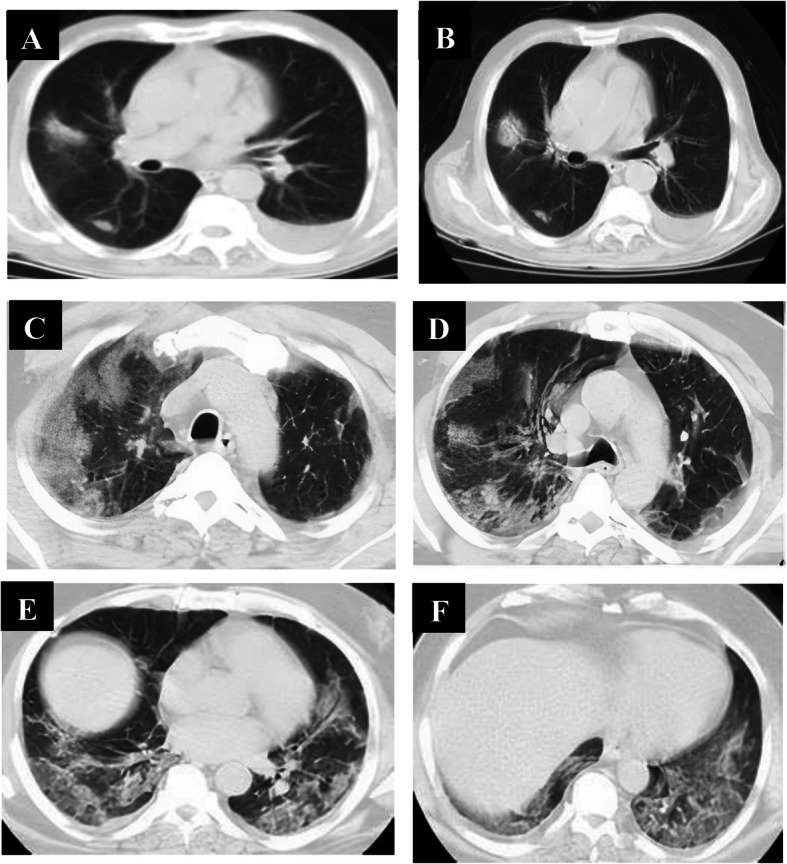
A 45-year-old male patient complaining of cough, fatigue, and sore throat; no respiratory distress (mild symptoms). Non-contrast axial chest CT (**a**–**d**) 9 days from onset of symptoms (late phase) revealed multiple extensive confluent patchy areas of ground opacities and consolidation seen scattered at both lungs; central and peripheral in distribution

All patients in the late phase group (8–14 days, *n* = 36) had positive CT findings. CT abnormalities were combined GGO and consolidation in 36 patients, crazy-paving in 24 patients, reversed halo in 20 patients, and linear consolidation in 28 patients, and peripheral distribution of the pulmonary lesions was noted in 4 patients and diffuse distribution in 32 patients. All those patients had bilateral pulmonary affection: 4 lobes of affection in 13 patients and 5 lobes of affection in 24 patients. Total lung severity score: score 17 in 4 patients, score 18 in 12 patients, score 19 in 12 patients, and score 20 in 8 patients (Fig. 3).

**Fig. 4:**
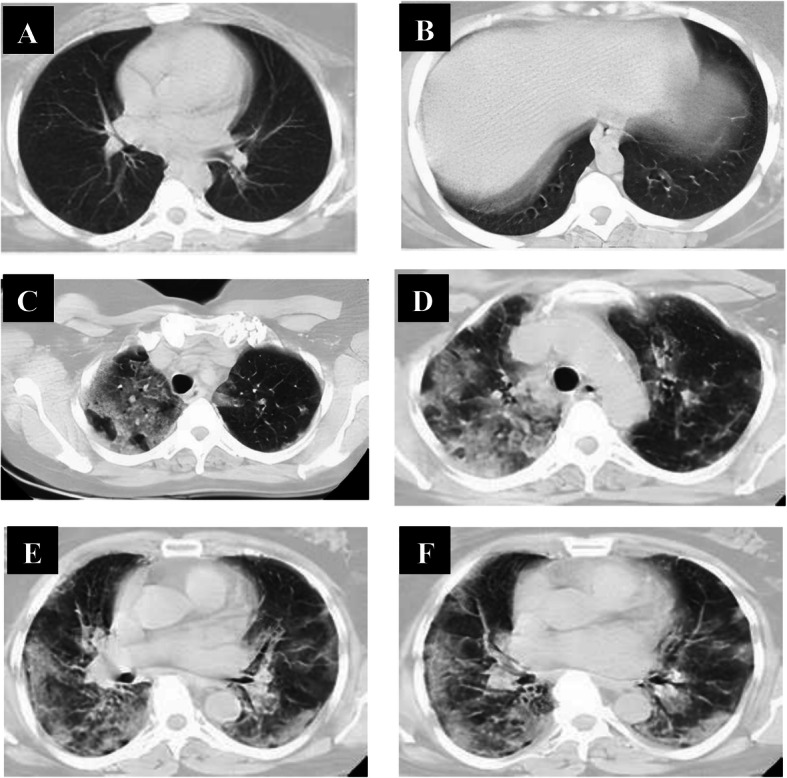
A 33-year-old male patient complaining of fever, sore throat, and cough (mild symptoms). Non-contrast axial chest CT (**a**, **b**) 2 days after onset of symptoms (early phase) revealed two patchy areas of consolidation seen at the right lung middle and lower lobes with air bronchogram and left-sided pleural effusion. With the persistence of the same symptoms (no respiratory distress), follow-up chest CT (**c**–**f**) 7 days from the onset of symptoms revealed more extensive lesions in the form of multiple confluent patchy areas of ground-glass opacities, consolidation, and crazy-paving seen scattered at both lungs: central and peripheral in distribution and bilateral minimal pleural effusion

**Fig. 5:**
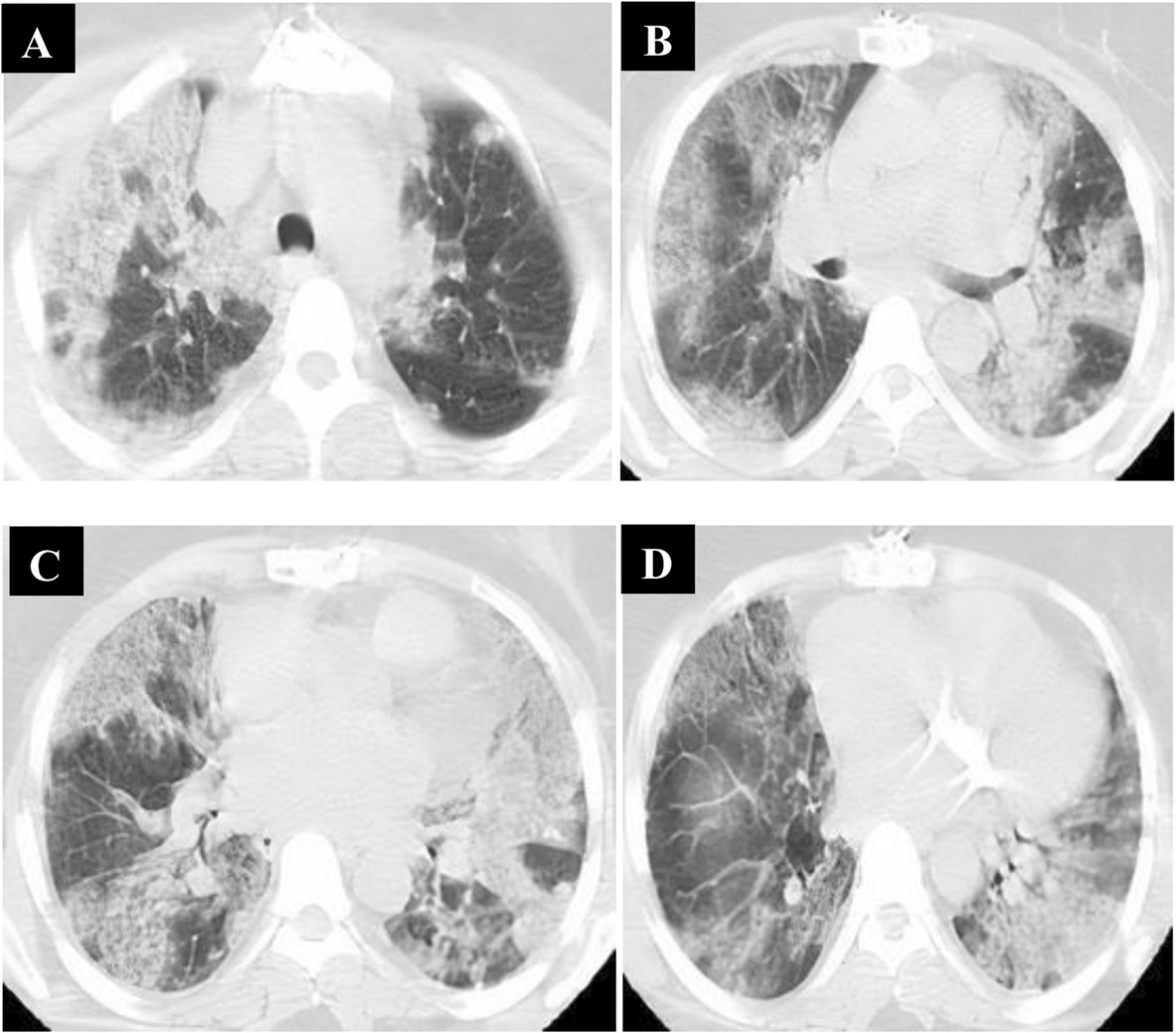
A 36-year-old female patient complaining of dyspnea, fatigue, fever, and cough (severe symptoms). Non-contrast axial chest CT (**a**, **b**) 1 day from the onset of symptoms (early phase) revealed normal CT study. With the persistence of the same symptoms, follow-up chest CT (**c**–**f**) 6 days from onset of symptoms revealed multiple extensive confluent patchy areas of ground-glass opacities and consolidation seen scattered at both lungs: central and peripheral in distribution

**Fig. 6:**
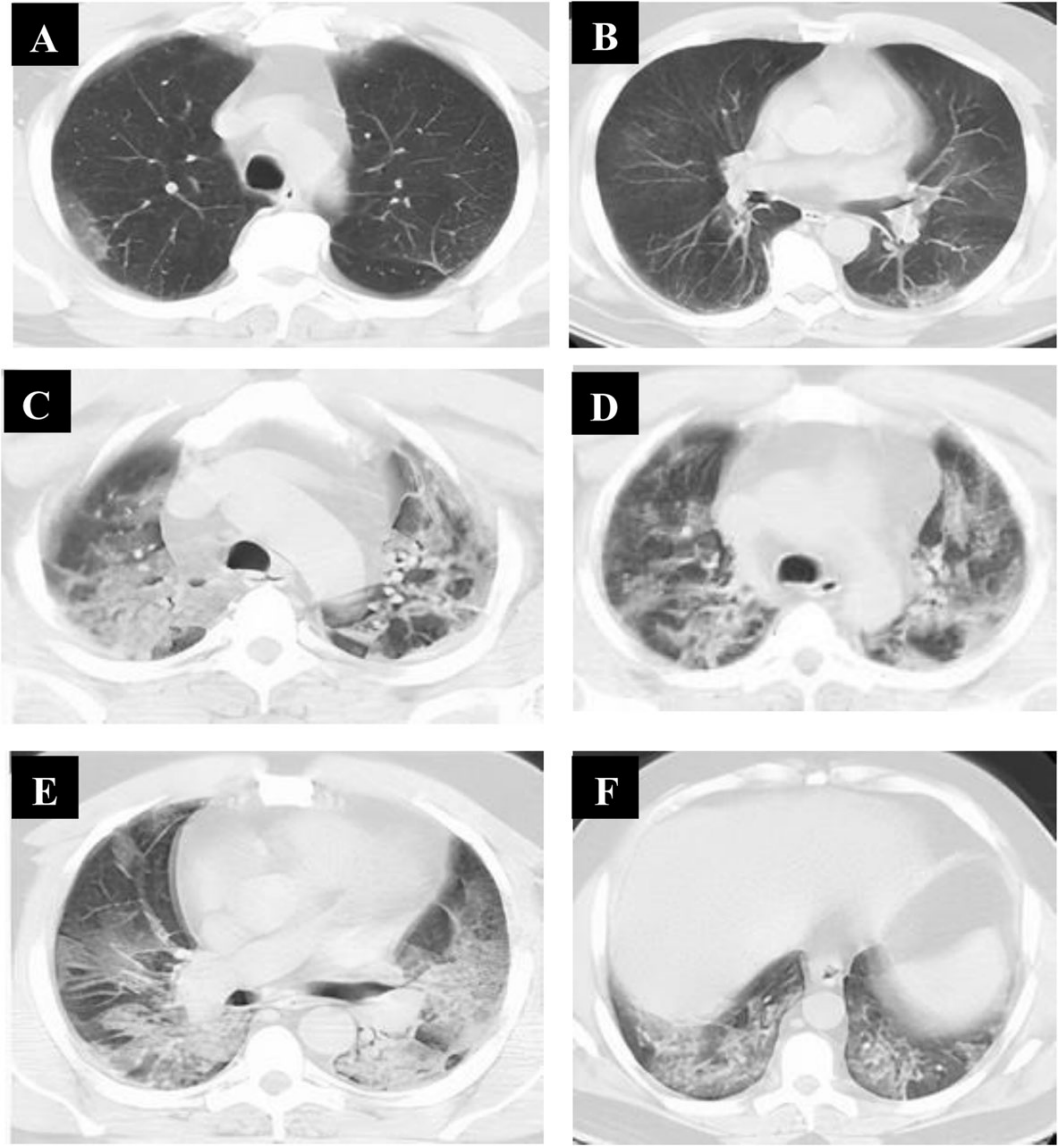
A 42-year-old female patient complaining of diarrhea and cough (mild symptoms). Non-contrast axial chest CT (**a**, **b**) 2 days from onset of symptoms (early phase) revealed few patchy areas of ground-glass opacities and consolidation seen at basal segments of both lungs lower lobes: peripheral in distribution. With the progression of symptoms (developed dyspnea), follow-up chest CT (**c**–**f**) 6 days from onset of symptoms revealed more extensive lesions in form of multiple confluent patchy areas of ground-glass opacities, consolidation, and crazy-paving seen scattered at both lungs: central and peripheral in distribution

Follow-up chest CT studies were done for patients in the early phase (*n* = 60) after 4–5 days from onset of symptoms, and we reported no CT lung abnormalities in 8 patients and positive CT findings in 52 patients. CT lung abnormalities were progressed than the initial CT: GGO only in 7 patients, consolidation only in 8 patients, combined GGO and consolidation in 38 patients, and crazy-paving in 4 patients. Peripheral distribution was noted in 8 patients and diffuse distribution in 44 patients. 52 patients had bilateral affection with two lobes of affection in 12 patients, three lobes of affection in 19 patients, 4 lobes of affection in 15 patients, and 5 lobes of affection in 6 patients. Total lung severity score: 8 patients with score 10, 4 patients with score 13, 25 patients with score 16, and 15 patients with score 17 (mean of 13.11 ± 5.657) (Figs. 4, 5, and 6).

**Fig. 7:**
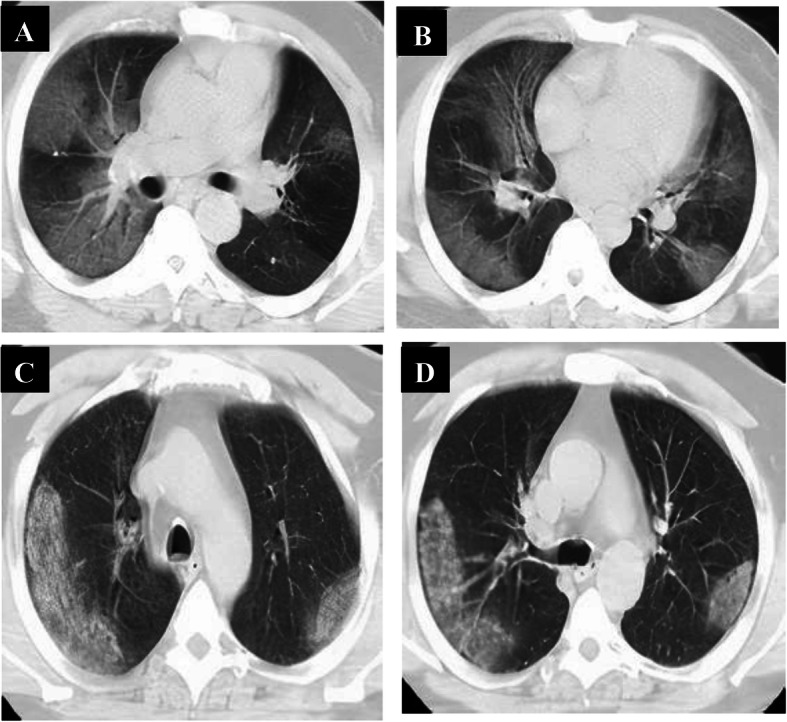
A 51-year-old male patient complaining of cough and dyspnea (severe symptoms). Non-contrast axial chest CT (**a**, **b**) 6 days from the onset of symptoms (intermediate phase) revealed multiple patchy areas of ground-glass opacity seen scattered at both lungs: peripheral in distribution. With the persistence of symptoms, follow-up chest CT (**c**, **d**) 9 days revealed multiple patchy areas of consolidation seen scattered at both lungs: peripheral in distribution

Follow-up chest CT studies were done for 108 patients in the intermediate phase after 7–8 days from onset of symptoms, and 8 patients were missed due to death. We reported positive CT findings in all of them; CT lung abnormalities were progressed than initial CT; GGO only in 9 patients, combined GGO and consolidation in 99 patients, crazy-paving in 14 patients, reversed halo in 8 patients, and linear consolidation in 32 patients. Peripheral distribution was noted in 12 patients, and diffuse distribution in 96 patients. 108 had bilateral affection with three lobes of affection in 30 patients, 4 lobes of affection in 41 patients, and 5 lobes of affection in 37 patients. Total lung severity score is as follows: 26 patients with score 13, 21 patients with score 15, 27 patients with score 16, 14 patients with score 18, and 20 patients with score 19 (mean of 15.89 ± 2.13) (Fig. 7).

The analysis of the chest CT findings in patients with mild symptoms showed negative CT findings in 12 patients and positive CT findings in 112 patients. CT abnormalities were GGO only in 12 patients, consolidation only in 7 patients, combined GGO and consolidation in 56 patients, crazy-paving in 52 patients, reversed halo in 32 patients, linear consolidation in 22 patients, and pleural effusion in 4 patients. Peripheral distribution was noted in 33 patients, and diffuse distribution in 79 patients. 112 patients had bilateral affection with 2 lobes of affection in 4 patients, three lobes of affection in 9 patients, 4 lobes of affection in 52 patients, and 5 lobes of affection in 47 patients. Total lung severity scores are as follows: 3 patients with score 4, 5 patients with score 6, 1 patient with score 7, 2 patients with score 13, 4 patients with score 14, 5 patients with score 15, 18 patients with score 16, 27 patients with score 17, 20 patients with score 18, 22 patients with score 19, and 5 patients with score 20.

Among patients with moderate to severe symptoms, 24 patients had negative CT findings and 64 patients had positive CT findings. CT abnormalities were GGO only in 21 patients, consolidation only in 5 patients, combined GGO and consolidation in 41 patients, crazy-paving in 4 patients, reversed halo in 4 patients, and linear consolidation in 6 patients. Peripheral distribution was noted in 18 patients, and diffuse distribution in 46 patients. 60 patients had bilateral pulmonary affection, and 4 patients had unilateral affection with one lobe of affection in 4 patients, 2 lobes of affection in 3 patients, three lobes of affection in 32 patients, 4 lobes of affection in 13 patients, and 5 lobes of affection in 12 patients. Total lung severity scores are as follows: 4 patients with score 2, 4 patients with score 3, 5 patients with score 4, 3 patients with score 6, 3 patients with score 7, 2 patients with score 13, 8 patients with score 14, 12 patients with score 15, 5 patients with score 16, 9 patients with score 17, 6 patients with score 19, and 3 patients with score 20. Analysis of pulmonary CT findings in studied patients according to the severity of symptoms is shown in Table 3.

## Discussion

In humans, coronaviruses are among the spectrum of viruses that cause the common cold and more severe respiratory diseases. The incoming coronavirus was named severe acute respiratory syndrome coronavirus 2 (SARSCoV-2) by the International Committee on Taxonomy of Viruses (ICTV). On February 11, 2020, the disease was denominated COVID-19 by the World Health Organization (WHO) [3, 6, 14]. It is stated to be the sixth public health emergency worthy of international attention. COVID-19 is highly contagious and has spread worldwide [26].

Management of the patient with COVID-19 is highly dependent on disease diagnosis. A specific viral nucleic acid assay using real-time reverse transcription-polymerase chain reaction (RT-PCR) was readily developed to confirm the diagnosis of COVID-19 and prohibit the spread of the COVID-19 epidemic [15, 16]. However, nucleic acid testing has tough laboratory specifications and demands a long time before the availability of results [27, 28].

Moreover, the absence of early abnormalities on chest radiographs can lead to a large number of false negatives. Thin-section chest CT is more sensitive than chest radiography, showing abnormal lung parenchymal changes in the early stages of the disease. It can detect abnormalities earlier than RT-PCR testing. So, high-resolution CT has been included as one of the main tools for screening, primary diagnosis, and evaluation of disease severity. The National Health Commission of the People’s Republic of China has promoted diagnosis depending on clinical and chest CT findings alone [29–33].

The diagnosis and treatment program (6th version) published by the National Health Commission of the People’s Republic of China had stated radiologic features diagnostic for viral pneumonia as important diagnostic criteria for COVID-19 that can exert a great role in the management of patients with suspected SARS-CoV-2 infection; mainly early isolation, particularly with the lack of scientifically proven therapies for the treatment of COVID-19 [14, 27, 28].

The main radiologic feature of acute insult is ground-glass opacities that may coalesce into dense consolidation and then gradually evolve and organize in often a more linear pattern confined mainly to the lung periphery and slightly with a crazy-paving pattern or evolution of a reverse halo sign [25].

Koo et al. [34] reported that confluent patches of GGO and consolidation distributed at peripheral parts of both lungs (subpleural) were two prime CT signs of COVID-19 pneumonia in their study and were confined to the middle and lower zones of the lung on the initial chest CT. Follow-up CT showed that with the advance of the disease, consolidation, and coalescing infiltrates intervened in the lungs affecting the upper lobes in some patients, all the five lobes of both lungs were affected giving white lung appearance. These pathologic changes and rapid progression in CT findings could be attributed to involving angiotensin-converting enzyme causing diffuse alveolar damage [35].

We noted predominant affection of peripheral lung zones (23 patients), unilateral lung affection (24 patients), three lung lobes of affection (13 patients), and lower severity score with a mean of (2.13) in the early phase. In the intermediate phase, there was a predominantly diffuse distribution of lung abnormalities (88 patients), bilateral lung affection (112 patients), and four lung lobes of affection (48 patients) with a mean severity score of 16.08. In the late phase, there was a predominantly diffuse distribution of lung findings (32 patients), bilateral lung affection in 36 patients, and five lung lobes of affection (24 patients) with a mean severity score of (17.83).

In the study performed by Dinga et al. [36], in the early stage of symptomatic COVID-19 (0–4 days), 21.2% of the CT scans showed no abnormalities. In case of CT abnormalities, peripheral GGO was the most important imaging manifestation (76.5%), indicating the disease may mainly invade the terminal respiratory bronchi or alveoli at first. Some patients also showed crazy-paving pattern (36.1%), consolidation (25.5%), and linear opacities (6.3 %) on the early stage CT, which is likely due to either rapid progress of the disease with poor prognosis or shorter course with good prognosis.

Also, Pan et al. [37] found in their study that CT features of the lesions were variable in the progressive stage (5–9 days). Crazy-paving pattern, consolidation, and linear opacities increased significantly, denoting interstitial edema and alveolar exudation. The frequency of crazy-paving pattern, consolidation, and linear opacities peaked at stage 3 (10–14 days), stage 4 (15–21 days), and stage 5 (22–28 days), respectively, and decreased later, reflecting changing patterns of pulmonary abnormality according to the stage and a long process of disease progression. Also, changes of chest CT in patients with COVID-19 at different stages may reflect the pathological changes to some extent.

A crazy-paving appearance, reversed halo sign, and linear consolidation were not detected during the early phase in the current study, and these changes were reported many days from onset of symptoms; more severe lung changes reported with longer time from the onset of the symptom correlate with the pathophysiology of the disease process. In the intermediate phase, crazy-paving is noted in 32 patients and reversed halo is noted in 16 patients. In the late phase, crazy-paving is noted in 24 patients, the reversed halo is noted in 20 patients, and the linear consolidation is noted in 28 patients.

Chest CT has limited sensitivity and negative predictive value early after symptom onset and is thereby not a reliable standalone tool to rule out COVID-19 infection. CT examination should be repeated for the high-risk population [25, 38]. This matches with our study; we reported no CT lung abnormalities in 32 patients in the early phase and 4 patients in the intermediate phase in initial CT studies with sensitivity 83%, specificity 100%, and negative predictive value 49.3%, and on follow-up, CT studies, 24 of them had positive findings in the early phase and all the followed patients had positive findings in intermediate phase with sensitivity 96.2%, specificity 100%, and negative predictive value 81.4%. There was a significant difference in CT severity score between different phases mainly between early and intermediate phases.

We found high total lung severity score in some patients with mild symptoms with a mean of 14.77 and low total lung severity score in some patients with moderate to severe symptoms with a mean of 9.14, and this was attributed to the time interval between the symptoms onset and the initial CT scan which were longer in some patients with mild symptoms than in others with moderate to severe symptoms indicating more relation of the lung abnormalities to duration than the severity of symptoms.

Exposure to radiation is an expected risk in the application of CT suggesting low-dose scan mode or techniques, especially for children and pregnant women. Cross-infection at the time of scan is another risk of apprehension that makes it necessary to take strict precautions during the scan process [39].

The main limitation of this study was difficult follow-up observations of lung changes of the disease at thin-section CT for patients in the late phase due to discharge or dismiss due to death. Also, no lung tissue biopsies were available to correlate between radiological and histopathologic findings.

## Conclusion

Thin-section chest CT is an essential modality for early detection of lung abnormalities in COVID-19 pneumonia and assessment of progression of lung lesions which correlated with the pathogenesis of disease and could be used as a screening modality. Lung abnormalities in COVID-19 pneumonia correlated with the duration of symptoms rather than the severity of symptoms.

## Data Availability

The authors confirm that all data supporting the finding of the study are available within the article, and the raw data and data supporting the findings were generated and available at the corresponding author upon request.

## Abbreviations

COVID-19: Coronavirus disease 2019
CRP: C-reactive protein
CT: Computed tomography
ESR: Erythrocyte sedimentation rate
RT-PCR: Reverse-transcriptase polymerase chain reaction
